# Efficient Allele-Specific Targeting of LRRK2 R1441 Mutations Mediated by RNAi

**DOI:** 10.1371/journal.pone.0021352

**Published:** 2011-06-21

**Authors:** Laura de Yñigo-Mojado, Itziar Martín-Ruíz, James D. Sutherland

**Affiliations:** Proteomics Unit, CIC bioGUNE, Derio, Bizkaia, Spain; National Institutes of Health, United States of America

## Abstract

Since RNA interference (RNAi) has the potential to discriminate between single nucleotide changes, there is growing interest in the use of RNAi as a promising therapeutical approach to target dominant disease-associated alleles. Mutations in the leucine-rich repeat kinase 2 (LRRK2) gene have been linked to dominantly inherited Parkinson's disease (PD). We focused on three LRRK2 mutations (R1441G/C and the more prevalent G2109S) hoping to identify shRNAs that would both recognize and efficiently silence the mutated alleles preferentially over the wild-type alleles. Using a luciferase-based reporter system, we identified shRNAs that were able to specifically target the R1441G and R1441C alleles with 80% silencing efficiency. The same shRNAs were able to silence specifically mRNAs encoding either partial or full-length mutant LRRK2 fusion proteins, while having a minimal effect on endogenous wild-type LRRK2 expression when transfected in 293FT cells. Shifting of the mutant recognition site (MRS) from position 11 to other sites (4 and 16, within the 19-mer window of our shRNA design) reduced specificity and overall silencing efficiency. Developing an allele-specific RNAi of G2019S was problematic. Placement of the MRS at position 10 resulted in efficient silencing of reporters (75–80%), but failed to discriminate between mutant and wild-type alleles. Shifting of the MRS to positions 4, 5, 15, 16 increased the specificity of the shRNAs, but reduced the overall silencing efficiency. Consistent with previous reports, these data confirm that MRS placement influences both allele-specificity and silencing strength of shRNAs, while further modification to hairpin design or MRS position may lead to the development of effective G2019S shRNAs. In summary, the effective shRNA against LRRK2 R1441 alleles described herein suggests that RNAi-based therapy of inherited Parkinson's disease is a viable approach towards developing effective therapeutic interventions for this serious neurodegenerative disease.

## Introduction

Parkinson's disease (PD) is a neurodegenerative disease causing movement disorders including bradykinesia, rigidity and resting tremors that are accompanied by pathological signs of dopamine neuron degeneration in the substantia nigra area of midbrain. Although most cases are thought to be sporadic, studies of familial PD have revealed a number of genes to have a causative and associated role in the disease. First characterized in genetic studies of affected families in England and in the Basque region in northern Spain [1], [2], mutations in leucine-rich repeat kinase 2 (LRRK2) have been identified as the underlying cause of up to 10% of familial PD cases and 2–3% of sporadic cases, with varying numbers and allele distribution depending upon the populations studied [3], [4]. LRRK2 is a complex 280 kD protein with multiple domains that mediate protein interactions and subcellular distribution, and possess enzymatic activity. Recent studies suggest an interplay between LRRK2 and other proteins that have been implicated with PD, including alpha-synuclein, parkin, and PINK-1 [5], [6], [7], [8]. At present, most evidence suggests that LRRK2 mutations cause an overall gain-of-function that leads to neurotoxic effects, consistent with the observed autosomal-dominant mode of inheritance.

In describing the molecular action of LRRK2, two domains with enzymatic activity have been of particular focus, namely the GTPase (termed ROC or Ras of complex proteins) and kinase domains. The most prevalent LRRK2 mutations affect the two domains involved with enzymatic activity, indicative of their functional importance. The originally described Basque mutation R1441G lies within the ROC GTPase domain, and more recently identified mutations affecting the same amino acid (R1441C, R1441H) have been described in affected PD patients [9], [10], [11]. There is ongoing controversy in the literature about the positive or negative effects of these mutations on GTPase activity [12], [13], [14] but what remains clear is that the ROC domain modulates kinase activity, LRRK2 folding and dimer formation [15], [16], [17], [18], [19]. Overall, the most prevalent LRRK2 mutation is G2019S and affects the serine-threonine kinase domain. From large population studies, the estimated prevalence of this mutation is 1% of global PD cases, 6% of inherited and 3% of sporadic PD cases in European populations, and up to 42% in North African populations [3], [4]. Functional studies suggest that this mutation leads to increased kinase activity, affecting both autophosphorylation [20], [21], [22], [23] and the phosphorylation of other substrates, such as the ezrin-radixin-moesin family of proteins [24]. This may, in turn, influence actin-related events in neurite outgrowth [25]. Both of these enzyme domains are therefore logical targets for drug-based therapeutic approaches [26], [27].

Another promising intervention approach to genetic diseases lies in the development and application of therapies based upon RNAi [28], [29], [30], [31], [32]. This conserved pathway uses short 21–25-mer double-stranded RNA molecules to regulate gene expression, through the control of mRNA stability and translation. Briefly, siRNAs are unwound, with one strand (the guide strand) being loaded into the RNA-induced silencing complex (RISC). The loaded RISC then scans the mRNA pool until finding a complementary target, leading to a cleavage of the target mRNA at a position corresponding to nucleotide position 10/11 of the guide strand [33]. When the mechanism underlying RNAi was initially described, it implied that short-interfering RNAs (siRNAs) may be able to discriminate between two sequences differing by a single nucleotide. Capitalizing on this capability, it may ultimately permit the exclusive targeting of only the disease-causing mRNAs, while leaving the corresponding wild-type mRNA unaffected to carry out its normal function. Indeed, recent studies have shown that allele-specific silencing by RNAi (ASP-RNAi) is possible and has been applied to disease-causing alleles linked to spinocerebellar ataxia [34], [35], [36], [37], Alzheimer's [38] and Huntington's disease [39], [40], [41], [42], and hereditary amyotrophic lateral sclerosis [40], [43], [44], [45], [46], [47], among others. While initial studies suggested that central positioning of the “mutant recognition site” (MRS) for ASP-RNAi allowed good discrimination, other studies have shown that off-center positioning showed improvements, especially in the case of purine:purine mismatches [40].

In this study, we have investigated whether ASP-RNAi may be a feasible approach for the treatment of LRRK2-based Parkinson's disease. Our results show that the G2019S allele may be somewhat resistant to ASP-RNAi and MRS positioning influences the balance between silencing strength and allele-specificity. In contrast, the R1441G/C alleles are efficiently identified and targeted when the MRS is centrally located. Since efficient targeting of particular mRNA sequences is likely dependent on multiple intrinsic and extrinsic factors (e.g. RNA secondary structure or positioning of RNA-binding proteins), development of efficient RNAi tools will depend on both informatic predictions and empirical testing.

## Results

**Figure 1:**
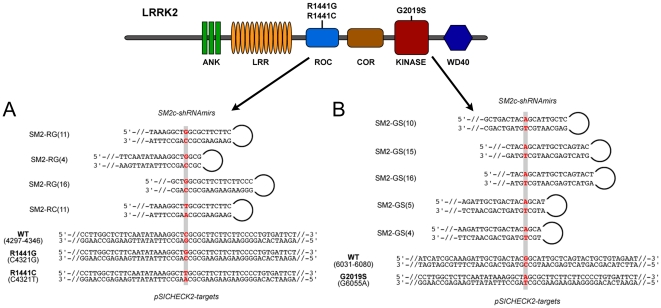
Overview of LRRK2 domains with shRNA and reporter template design. LRRK2 is a large protein (280 kD) composed of multiple domains [51]. Although PD-linked mutations have been described throughout the protein, only mutations addressed in this work that affect the ROC and kinase domains are shown. A. Schematic design of shRNAs to target ROC-associated mutations is shown, illustrating the MRS shifts and the nomenclature used. Numbering is according to 5′ to 3′ position in the antisense “guide” strand. The shRNAs were expressed from U6 promoter-based SM2c, and a post-Drosha/pre-Dicer version is depicted. Portions of the WT and mutant LRRK2 target sequences are shown, which were centrally located in a stretch of 400 bp included in the pSICHECK-2 vectors. B. The design of shRNAs and templates to target the G2019S kinase mutation is shown. ANK: Ankyrin-like, LRR: Leucine-rich repeats, ROC: Ras of Complex proteins, COR: C-terminal of ROC, WD40∶40 amino acid WD (or beta-transducin) repeats.

To induce RNAi, we decided to use short hairpin RNA (shRNA) as our model system. Although many advances have been made in the packaging and delivery of siRNAs across the blood-brain barrier [30], [32], shRNAs can be expressed transiently to test their efficacy and stability for long-term silencing, and can be tailored for potential use in gene- or cell-based therapy. Of the various vector configurations available, we chose to use the pSM2c-shRNAmir system [48], which is able to generate siRNA from shRNA more efficiently than other systems due to native processing by Drosha and Dicer enzymes of the miR30-based transcript into which the shRNA is embedded. Suitable control vectors were also available that efficiently silenced exogenous genes such as GFP. shRNAs were designed as 97-mer oligonucleotides, amplified by PCR and cloned into pSM2c (Figure 1).

For the proposed studies, we used the psiCHECK-2 vector (Promega) as a reporter system. The target sequence of choice is placed within the 3′UTR of an expression cassette of Renilla luciferase (Rluc; from *Renilla reniformis*), such that Rluc activity (measurable by luminescence assay) is correlated with the silencing potential of the test shRNAs. Also located within the same plasmid is an internal control cassette encoding firefly luciferase (FFluc; derived from *Photinus pyralis)*. We used this system to clone short 400 bp fragments of the human LRRK2 gene that encompassed our region of interest (Figure 1). Ten different constructs were prepared: four were taken from the DNA encoding the ROC domain, five from the kinase domain, and one from a distinct site in LRRK2 (SM2-656; not shown in schematic diagram).

### Targeting of LRRK2 R1441G mutation is effective with central MRS

**Figure 2:**
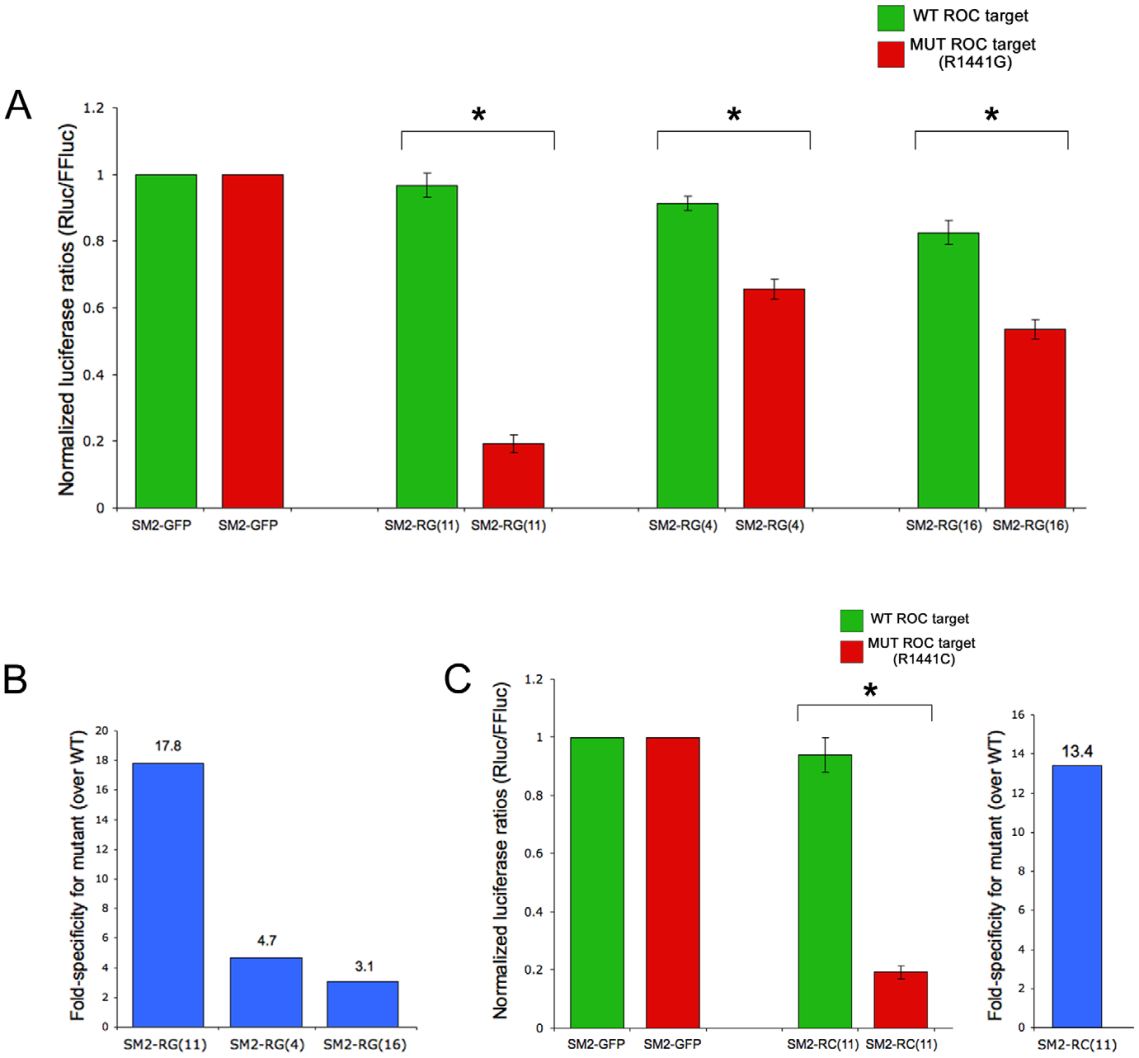
ASP-RNAi against R1441 LRRK2 alleles is effective when MRS is centrally located. A. Luciferase-based assays demonstrated that shRNA SM2-RG(11) is quite selective in recognizing mutant over wild-type LRRK2, and leads to potent silencing of the mutant target. Shifting of the MRS either 5′ or 3′ within the guide strand reduced both specificity and silencing strength of the resulting shRNAs. * denotes a p-value of <0.01; n.s. =  not significant. B. A representation of fold-specificity is shown, calculated by comparing silencing strength of shRNA towards mutant templates versus wild-type template (for example, 81% knockdown of R1441G by SM2-RG(11) compared to 4.5% knockdown of wild-type R1441 results in 17.8-fold specificity). C. Similar experiments using a distinct shRNA and template to test R1441C ASP-RNAi showed that placing the MRS at position 11 is also effective for a different R1441 allele.

Transient transfections into 293FT cells were performed with combinations of shRNA expression plasmids and luciferase reporter targets. To determine specificity, shRNAs were tested against wild-type and mutant targets and results were normalized to FFLuc and those obtained with a non-targeting shRNA (SM2-GFP). For targeting LRRK2 R1441G, we designed three shRNA constructs that varied the position of the MRS. As shown in Figure 2, we observed the best combination of silencing strength and allele-specificity when the MRS was placed at position 11 [shRNA SM2-RG(11)]. Although weak recognition of the wild-type allele was seen, the R1441G allele was targeted with 17.8-fold higher efficiency for an overall silencing strength of 80%. Shifting of the MRS to the “seed” region [position 4; SM2-RG(4)] or position 16 [SM2-RG(16)] reduced both specificity and silencing strength towards the R1441G target (4.7-fold and 3.1-fold, 35% and 49%, respectively).

### Targeting of LRRK2 R1441C mutation is equally effective

The R1441 residue of LRRK2 is a hotspot for mutation, with Gly/Cys/His variants described to date. This suggests an important structural and/or regulatory role for R1441, which is contained within the ROC GTPase domain. Based upon our evidence that MRS position 11 was effective for targeting the R1441G allele, we prepared an additional shRNA and luciferase target corresponding to R1441C. As shown in Figure 2C, SM2-RC(11) efficiently targeted the R1441C reporter (81% overall silencing strength) and moreover distinguished it from the wildtype reporter (13.4-fold specificity). In addition, the mutant R1441G reporter was poorly targeted by SM2-RC(11) (data not shown). Based on these results, we conclude that locating the MRS at position 11 optimizes the specific discrimination of R1441 alleles.

### Targeting of LRRK2 R1441G mutation is effective using additional reporters

**Figure 3:**
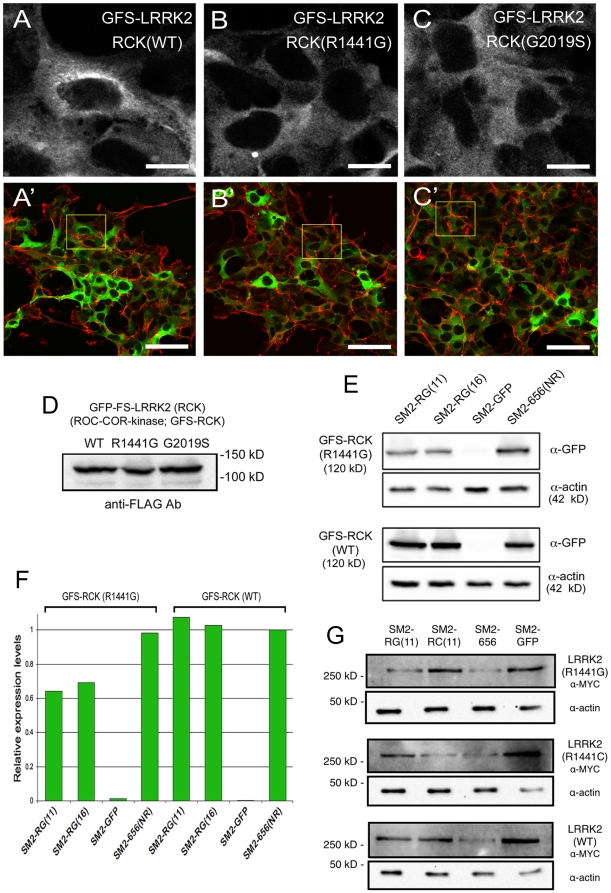
ASP-RNAi using SM2-RG(11) is also effective using a different reporter system. A–D. Stable cell lines expressing the RCK domains of LRRK as GFS-fusions showed similar cytoplasmic localization of the fusion proteins and similar expression levels. A'–C' show F-actin counterstaining with Alexa568-phalloidin, with regions depicted in A–C highlighted by a yellow box. RCK: ROC-COR-kinase, GFS: GFP-FLAG-STREP. Scale bars: 5 µm A–C, 25 µm A'–C'. D. GFS-LRRK2(RCK) fusions (WT and variants) have expected size and are expressed at similar levels, as detected using FLAG antibody. E–F. Western blot analysis of cells treated with the indicated shRNAs showed that SM2-RG(11) and SM2-RG(16) are similarly effective at recognizing and reducing expression the GFS-RCK(R1441G) fusion protein. An shRNA targeting a non-relavant LRRK2 sequence [i.e. outside of the RCK region, SM2-656(NR)] was used a negative control and, in conjunction with the GFS-RCK(WT) target, was used as a reference for determining relative expression levels. GFP antibody was used to detect the GFS-LRRK2(RCK) fusions and band intensities were normalized to actin. G. ASP-RNAi by SM2-RG(11) and SM2-RC(11) on exogenous full-length LRRK2. Combinations of shRNAs and 2xMYC-tagged LRRK2 (WT or mutants) were transfected into 293FT cells and analyzed after 48 hrs by western blotting with MYC antibody, with actin shown as a loading control.

Because LRRK2 is encoded by a long mRNA transcript (Refseq NM_198578.3; 9239 bp; ORF 7584 bp), mRNA secondary structure could be a contributing factor in targeting efficiency of shRNAs. We therefore tested longer targets using a different reporter context. We used truncated GFP-fusions as additional reporters for gene-silencing that would display LRRK2 mRNA structure in a more native context. We generated three GFS-fusion constructs encompassing the ROC-COR-KINASE (RCK) domains of LRRK2, containing 2517 bp of LRRK2 sequence, either as wild-type or with mutations (R1441G in the ROC domain or G2019S in the kinase domain), and used them to establish stable 293FT cell lines. The GFS-RCK fusions were localized diffusely throughout the cytoplasm and excluded from the nucleus, and mutations did not alter the pattern. Expression levels of the transgene between the three cell lines were comparable and the fusion proteins were detectable with FLAG or GFP antibodies (Figure 3A–E).

We tested SM2-RG(11) and SM2-RG(16) for targeting of GFS-fusion reporters and they yielded similar results of moderate silencing strength and good specificity (Figure 3E–F). Compared to the luciferase-based assay, SM2-RG(11) displayed reduced silencing strength but maintained high specificity (40%, 19.8-fold; respectively), whereas SM2-RG(16) resulted in slightly reduced silencing strength but increased specificity (35%, 17.2-fold; respectively). The negative control SM2-656(NR), designed to target a distinct LRRK2 sequence not included in the RCK-encoding region, had little effect on either GFP-fusion, whereas the positive control SM2-GFP potently silenced both. We still wondered whether ASP-RNAi was possible in full-length LRRK2, so we obtained (or generated) MYC-tagged constructs for testing of the SM2-RG(11) and SM2-RC(11) shRNAs. Although we were unable to perform quantitation on blots from these experiments due to background, it is qualitatively apparent that each allele-specific shRNA is able to silence its cognate target in the full-length context, with reduced or no effect on non-cognate targets (Figure 3G). The positive control SM2-656 shRNA can silence all LRRK2 variants (WT, R1441G, and R1441C), whereas the negative control SM2-GFP has no effect. Taken together, these results demonstrate the importance of using different reporter systems and support our conclusion that effective R1441 allele-specific targeting can be accomplished.

### Targeting of LRRK2 G2019S mutation is less effective

**Figure 4:**
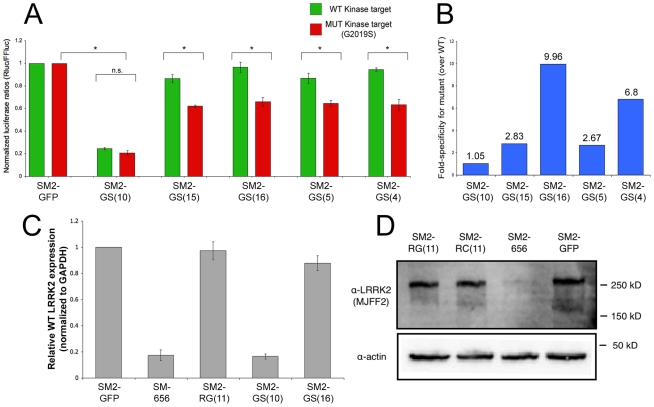
ASP-RNAi against G2019S LRRK2 alleles is most effective when MRS is not centrally located. A. Luciferase-based assays demonstrated that shRNA SM2-GS(10) has potent silencing capability but cannot discriminate between mutant and wild-type G2019 alleles. Shifting of the MRS either 5′ or 3′ within the guide strand increased the specificity of resulting shRNAs, but reduced their overall silencing strength. * denotes a p-value of <0.01. B. A representation of fold-specificity is shown, calculated by comparing silencing strength of shRNA towards mutant template versus wild-type template. Average values were used for comparison. SM2-GS(16) was the best shRNA tested in this study in the compromise between specificity and silencing strength, but only slightly better that SM2-GS(4). C. Quantitative real-time PCR analysis of selected shRNAs shows that the effect of SM2-RG(11) and SM2-GS(16) on endogenous wild-type LRRK2 in 293FT cells is weak (3% and 12%, respectively) and therefore these are promising shRNAs for developing ASP-RNAi for the associated mutant alleles. D) Endogenous LRRK2 protein is only weakly silencing by SM2-RG(11) and SM2-RC(11) shRNAs, compared to the SM2-GFP control. A positive control, SM2-656, which targets a distinct region of WT LRRK2, is effective at silencing. LRRK2 was detected by immunoprecipitation, and actin is detected in input fractions.

Since its description as a gene underlying familial PD, multiple alleles have been described in LRRK2, with the most prevalent being G2019S, which is located in the kinase domain. This allele is associated with 4–6% of cases of inherited PD [3], [4]. We initially designed 3 shRNAs to target the G2019S allele, varying the MRS position to assess effects on silencing specificity and strength (see Figure 1 for schematics). When tested using luciferase-based reporters, a centrally located MRS [SM2-GS(10)] was able to induce potent silencing of the LRRK2 template (80%), but was not able to discriminate between the wildtype and G2019S alleles (Figure 4A–B). Outward shifting of the MRS, either towards position 5 (the “seed-region” area; nomenclature derived from microRNAs) or towards position 15, led to an increase in the allele-specific recognition of G2019S (up to 2.8-fold), but also led to a decrease in silencing strength (35%).

We tested an additional shRNA with the MRS in position 4 [SM2-GS(4)] and it exhibited a slight increase in specificity over SM2-GS(5), demonstrating that stepwise moves in MRS can alter shRNA properties. A recent study focused on ASP-RNAi optimization using siRNAs suggested that position 16 was particularly suited for locating the MRS, especially in the case of purine-purine mismatches [40]. For this reason we designed and tested an additional shRNA [SM2-GS(16)] to determine whether any improvements could be observed. Although the silencing strength remained moderate (34%), this shRNA exhibited the best specificity for G2019S (almost 10-fold over WT) of the five shRNAs tested. We used the GFS-RCK cell lines to test SM2-GS(10) and SM2-GS(16) and results correlated with the luciferase assays, and furthermore, similar luciferase results were obtained when co-transfections were followed by transient puromycin selection (to confirm presence of SM2 plasmids) (data not shown). While the SM2-GS(16) is promising, we conclude that efficient allele-specific silencing of the G2019S allele is problematic and will require the testing of additional hairpin designs or switching to synthetic siRNA approach.

Finally, we tested whether selected shRNAs had any effect on the expression of endogenous LRRK2 expression. (Figure 4C). Using quantitative real-time PCR, we found that LRRK2 mRNA is expressed at moderate levels (compared to GAPDH) in 293FT cells (derived from human embryonic kidney). Perhaps this is not surprising since that, in addition to neural tissues, LRRK2 is expressed in non-neural tissues such as lung, spleen, heart and notably, the kidney [49], [50]. A positive control shRNA (SM2-656) showed >80% reduction of LRRK2 expression in 293FT cells (a similar reduction was observed using this shRNA and a matched cognate template by luciferase reporter assay; data not shown). Consistent with results obtained by luciferase assay, we observed that the shRNA SM2-GS(10) strongly silences endogenous LRRK2 (83%), even with a mismatch in place. On the contrary, the best candidate shRNAs for R1441G [SM2-RG(11)] and G2019S [SM2-GS(16)] showed only slight reduction of the endogenous LRRK2 mRNA (3% and 12%, respectively). We also tested whether these shRNAs could reduce amount of endogenous LRRK2 protein by immunoblotting. Perhaps due to its size or solubility, levels of LRRK2 protein were exceeding low in cell lysates, however successful detection of LRRK2 in 293FT cells was possible after immunoprecipitation. Using this strategy, we show that, while SM2-656 can effectively reduce endogenous LRRK2 levels, SM2-RG(11) and SM2-RC(11) only weakly affect endogenous LRRK2 levels compared to a negative control (SM2-GFP). Taken together, these results further support the utility of these shRNAs for allele-specific applications.

## Discussion

### LRRK2 and RNAi-based therapies of neurodegenerative diseases

Mutations in LRRK2 are the most common cause of familial PD and also appear in sporadic cases of idiopathic PD [3], [4]. Initial studies uncovered the R1441-type and G2019S mutations that affect the ROC GTPase and kinase domains (respectively), which led us to focus on experiments designed to enhance our understanding of the effect of these mutations on LRRK2 function. These mutations act dominantly and most likely cause enzymatic or structural gain-of-function that lead to neuronal toxicity [51]. The two enzymatic domains may be inter-dependent, with studies suggesting that GTPase activity influences kinase activity [12], [14], [52], [53]. In the case of the GTPase domain, structural studies suggest that R1441 mutations could destabilize dimer formation [16], although it is unclear whether GTPase activity is linked to dimer formation [17]. Regarding the kinase, most reports show an increase in kinase activity as a result of the G2019S mutation [24], [54], [55]. LRRK2 itself is a substrate, and autophosphorylation or phosphorylation of adjacent LRRK2 dimers could have functional consequences, such as affecting its GTPase activity or binding to partners such as 14-3-3 and MKK3/6/7 proteins [21], [22], [56], [57]. LRRK2 kinase can target proteins of the ezrin/radixin/moesin (ERM) family, which function as cross-linkers between the plasma membrane and actin filaments, and LRRK2-induced phosphorylation of ERMs can impair neurite outgrowth [24], [25]. While the GTPase and kinase domains may be amenable to targeting by small-molecule enzymatic inhibitors [26], similar domains in other proteins may be secondarily targeted. Also, enzyme inhibitors will likely target wild-type LRRK2 as well. For this reason, we explored the use of ASP-RNAi as a method for targeting the dominant disease-causing alleles of LRRK2.

To our knowledge, the present study is the first that rigorously explores ASP-RNAi approaches to a gene linked to PD, with the hopes of developing efficient therapeutic interventions. There have been previously published reports of effective allele-specific siRNAs and shRNAs against genes linked to other neurodegenerative diseases, emphasizing the current interest and feasibility of this approach. Some examples include: ataxin 7 (spinocerebellar ataxia type 7; [37]), ataxin 3 (Machado-Joseph disease; [36], [58]), amyloid precursor protein APP (Alzheimer's disease; [59], [60], [61], [62]), prion protein (Creutzfeldt-Jakob disease; [63]), and huntingtin (Huntington's disease; [40], [42], [64], [65], [66]) and the Cu/Zn superoxide dismutase gene SOD1 (hereditary amyotrophic lateral sclerosis ALS; [40], [44], [45], [46], [67], [68], [69]).

Because of the difficulties associated with designing effective ASP-RNAi against disease-causing gene alleles, other approaches have also been proposed. Instead of designing highly specific shRNAs that can discriminate between the wild-type and mutant alleles, some investigators silence both alleles with the best shRNA that can be identified while simultaneously supplying a cDNA encoding a RNAi-resistant version of the gene of interest [35], [47]. Due to the size of the cDNAs and difficulty of controlling endogenous expression levels, this may not be ideal for some genes, especially LRRK2. It has also been proposed that silencing of both wild-type and mutant alleles may be tolerated and is perhaps beneficial, such as the case with huntingtin [70]. Recent knockout mouse studies suggest that LRRK2 may be dispensable for development and maintenance of dopaminergic neurons (which undergo degeneration in PD), but LRRK2 −/− mice suffer from renal defects linked to accumulated alpha-synuclein and increased apoptosis [71], [72]. Therefore a “non-allele-specific” RNAi approach may be feasible for LRRK2 if restricted to neural tissues. These examples serve to illustrate that research into the application of RNAi to neurodegenerative diseases is active, effective, and evolving.

### Efficient ASP-RNAi of LRRK2 for R1441 alleles

Since the first reports linking LRRK2 to PD, up to 80 mutations have been described in studies encompassing >1000 families/sporadic cases. We have focused on three of the most prevalent - and best characterized of the mutations, which affect the ROC domain (R1441G/R1441C) and the kinase domain (G2019S). Unlike many of the LRRK2 mutations previously reported, these three mutations have been linked to PD pathogenicity through segregation analysis [4]. Our results show that these mutations, especially those affecting R1441, are amenable to ASP-RNAi.

As seen in previous studies from others [40], [63], we have found that placement of the MRS within the siRNA/shRNA can influence both the silencing strength and specificity. In our case, a centrally located MRS in SM2-RG(10) was able to silence LRRK2 mutant R1441G better than wild-type (almost 18-fold better) with an overall silencing strength of 80%. By keeping the MRS in the same position, similar efficiencies were obtained for an shRNA targeting R1441C. Both of these mutations are caused by underlying missense mutations of the same nucleotide (protein change R1441G is caused by cDNA change c.4321C>G; R1441C is caused by c. 4321C>T). A third mutation affected the same amino acid (R1441H) has been described, suggesting that R1441 is a mutational “hotspot” [11]. Although we might expect that R1441H would also be efficiently targeted, it is caused by a mutation of a distinct adjacent nucleotide in the cDNA (c.4322G>A), so the efficiency of targeting this site by ASP-RNAi may also be distinct. In the case of R1441G, we have shown that shifting the MRS away from the central location caused a drop in both specificity and silencing strength.

In this study, we have used distinct reporter systems in cell-based assays and note that SM2-RG(11) performed better in the luciferase-based assay than the GFS-fusion-based assay (compare Figures 2 and 3). While this may be linked to various experimental factors, including length of LRRK2 sequence included in the reporter, time elapsed before analysis of silencing, and sensitivity of detection, it underscores the need to test shRNAs empirically by multiple assays. We also show that ASP-RNAi of R1441 alleles also works on full-length mRNAs. Importantly, the SM2-RG(11) shRNA has minimal effect on endogenous LRRK2 as judged by quantitative RT-PCR, while both RG(11) and RC(11) have minimal effect on endogenous LRRK2 protein levels. Thus, SM2-RG(11) is a good candidate to proceed into more physiological studies, perhaps in a recently described mouse model carrying the human R1441G LRRK2 allele [73], or in patient-derived cell lines made feasible by induced pluripotent stem cell technology [74].

### ASP-RNAi of the LRRK2 G2019S allele is less efficient

Our attempts to develop shRNAs against LRRK2 G2019S yielded a pair of shRNAs [SM2-GS(4) and SM2-GS(16)] that showed good discrimination of the mutant allele (7-fold to 10-fold over wild-type, respectively). However, these shRNAs showed only low-to-moderate silencing strength (around 30%). A centrally-located MRS gave more potent silencing, but showed no discrimination between the mutant and wild-type alleles. As mentioned earlier, in a recent study using synthetic siRNAs, position 16 for placement of the MRS has been shown to be a favorable position for enhanced allele-selectivity, especially in cases of purine-purine mismatches [40]. In the case of G2019S, which is caused by a mutation in the cDNA of c.6055G>A, the effect of the purine-purine mismatch is evident and position 16 leads to the highest allele-specificity of the 5 shRNAs tested. In the case of R1441G, which has a pyrimidine-purine mismatch c.4321C>G, we observed that placing the MRS in position 16 maintained allele-specificity, but position 11 was superior. Both examples are consistent and in agreement with the previous study [40], however we still believe that bioinformatic predictions need to be tested empirically and reported. This, in turn, will lead to better predictions.

Until more is known about the function of LRRK2 and whether a 30% reduction of the G2019S allele would be enough to improve the pathological outcome, efforts to further optimize shRNAs to target this allele are important. It has been reported that homozygous carriers of G2019S are indistinguishable in PD clinical characteristics from heterozygous carriers [75], suggesting that the mere presence rather than dosage of the G2019S allele is perhaps more important for the abnormal phenotype. Matters are further complicated by reports of incomplete penetrance, i.e. that some G2019S carriers (identified amongst control individuals) fail to exhibit symptoms of PD (reviewed in [4]). Certainly there is more sequence space to explore within the “window” surrounding the G2019S and perhaps a distinct MRS position will give the desired high-specificity, potent shRNA. Another solution may be to introduce additional mismatches beyond the mutation of interest, which has been shown to enhance ASP-RNAi in a recent study, especially in the case of shRNAs [63].

In conclusion, we believe that effective shRNAs are feasible for the G2019S, the most prevalent LRRK2 allele, and may be able to reach similar efficiencies to the R1441G and R1441C shRNAs we have described here. While RNAi-based therapies for neural tissues still face important issues such as delivery and cell-specific targeting (reviewed in [28], [29], [30], [31], [32]), ASP-RNAi offers a promising therapeutic approach to these as yet incurable diseases.

## Materials and Methods

### Plasmid construction for shRNAs and reporters

All shRNA expression plasmids were based on the retroviral vector SM2c (OpenBiosystems; [48]). Long single-stranded oligos containing hairpin-encoding sequences were amplified by PCR using Phusion high-fidelity polymerase (Finnzymes), digested with Xho1-EcoR1, and cloned into SM2c. As this vector is prone to recombination, all plasmid preparations were checked by agarose gel electrophoresis and sequenced to verify correct structure. Using a human LRRK2 cDNA (provided by J. Perez-Tur; CSIC, Valencia [1]), a fragment encompassing the RCK-COR-KINASE domains was PCR amplified and cloned as Not1-BamH1 fragments into LL-GFS-puro (a modified version of Lentilox 3.7 [76]), which includes an N-terminal GFP-FLAG-STREP tag and IRES-puro sequences. (Note: Lentilox 3.7 was provided by L. van Parijs, MIT; map available by WWWsearch; the U6-RNAi cassette has been removed; details of the plasmid construction available upon request). LRRK2 mutations (R1441G, G2019S) were similarly prepared in LL-GFS-puro by two-step overlapping PCR. For luciferase-based targets, sequences were amplified by PCR and cloned as Xho1-Not1 fragments in pSICHECK2 (Promega). For full-length LRRK2 targets, we obtained 2xMYC-LRRK2 (WT and R1441C) from Addgene (courtesy of M. Cookson). To obtain 2xMYC-LRRK2(R1441G), quickchange site-directed mutagenesis using Phusion polymerase was performed using oligos 469/470. All final constructs were verified by DNA sequencing (Stabvida, Portugal). A list of oligos used for these constructions is provided (Table S1).

### Luciferase assays and analysis

All assays were performed in human embryonic kidney-derived 293FT cells (Invitrogen) cultured in DMEM supplemented with 10% FCS, glutamine, and antibiotics. Transfections were performed in 24-well plates using Arrest-In reagent (OpenBiosystems), plus 400 ng of SM2c and 100 ng of pSICHECK2 target plasmids. Lysates were prepared 48 hours after transfection and measured using Dual-Luciferase reporter assay (Promega) in 96-well plates by a luminometer (Turner Biosystems, TD20/20). All values for Renilla luciferase (linked to RNAi activity) were normalized to firefly luciferase (internal control). Student t-tests were performed to determine significance. Final values are compiled from triplicate-well assays performed at at least twice. Additional controls included SM2c-GFP (OpenBiosystems), which expresses a potent shRNA targeting GFP, but gave negligible activity against LRRK2 targets. To evaluate silencing strength and specificity against mutant and wild-type targets, all SM2c hairpins targeting LRRK2 were compared to a non-relevant target corresponding to LRRK2 1-400.

### Stable cell line production, shRNA treatment and analysis

Lentivirus was prepared by calcium phosphate transfection of 293FT using LL-GFS-LRRK2 plasmids and psPAX2/pVSV-G [provided by D. Trono (EPFL, Lausanne) and L. van Parijs (MIT)] for packaging. Filtered supernatants were titered and used to infect 293FT cells (MOI 5) and stable populations were selected by puromycin treatment (0.5 µg/ml). For microscopic analysis, cells were plated on poly-L-lysine-coated coverslips, counterstained with Alexa568-phalloidin (Invitrogen) and imaged on a Leica TCS SP2 confocal microscope. Using SM2 plasmids (2 µg), cell lines were transfected using Arrest-In in 6-well plates. After 72 hours, cells were lysed directly in 1x SDS sample buffer and analysed by 10% SDS-PAGE and by immunoblotting using FLAG (Sigma M2 mAb 1∶3000) or GFP antibody (Santa Cruz sc8334; 1∶1000). Chemiluminesent signals were developed by SuperSignal West Pico reagent (Pierce), and captured and quantified by Chemidoc system and Quantity One software, respectively (Biorad). Excel, Photoshop and Illustrator software (Microsoft; Adobe) was used for statistical analysis and image preparation.

### Quantitative real-time PCR analysis

Transfections were performed (2 µg DNA, 6-well) and cells were collected after 48 hours. Total RNA was isolated using mi-Total (Metabion), and cDNA was prepared using Thermoscript reverse transcription (Invitrogen). Primer sequences were obtained from qPrimerDepot (http://primerdepot.nci.nih.gov) and primer efficiencies tested by sample dilution method. PCR reactions were prepared using iQ SYBR Supermix (Biorad) and analyzed using an iCycler (Biorad). Cycling conditions were 94°C 3′, (94°C, 15″; 57°C 15″; 72°C 30″; 40x), followed by a melt-curve analysis. The ΔΔCt method was used to calculate relative expression levels of LRRK2. The data presented is derived from triplicate PCRs of cDNA from three independent experiments.

### Endogenous LRRK2 detection by western blot

The shRNA transfections into 293FT were performed (10 µg DNA, 10 cm dish) and cells were treated after 24 hours with puromycin (0.5 µg/ml) to enrich for transfected cells. 72 hours after transfection, cells were lysed in 500 µl M-PER (Pierce) supplemented with protease inhibitors (Roche) and incubated at 1 hr, 4°C with agitation. After clearing by centrifugation, protein concentration was checked by Bradford reagent (Biorad) and 500 µg of protein was used in each immunoprecipitation. Inputs were saved as controls. LRRK2 mAb (Epitomics MJFF2; 5 µl) was added to lysate on ice for 30 minutes, before adding 30 µl of washed Gammabind Plus Sepharose (GE Healthcare) for 4 hrs, 4°C with agitation. After 5 washes with M-PER supplemented with 100 mM NaCl, beads were resuspended in sample buffer, analyzed by 6% SDS-PAGE (controls by 10%), transferred to PVDF membrane, and blocked in 5% nonfat milk, 0.1% Tween-20 in PBS. Blots were probed using LRRK2 (MJFF2; 1∶1000) or actin (Sigma AC74, 1∶5000), with IgG-light chain-specific secondary antibodies (Jackson Immunoresearch). LRRK2 signals were developed using SuperSignal West Femto reagent (Pierce).

## Supporting Information

Table S1 Oligonucleotides used in this study.
(TIF)
