# Supplementary figures and images for: Efficient Allele-Specific Targeting of LRRK2 R1441 Mutations Mediated by RNAi
Source: PLoS One. 2011 Jun 21;6(6):e21352. doi: 10.1371/journal.pone.0021352 (PMC3119704; doi:10.1371/journal.pone.0021352)

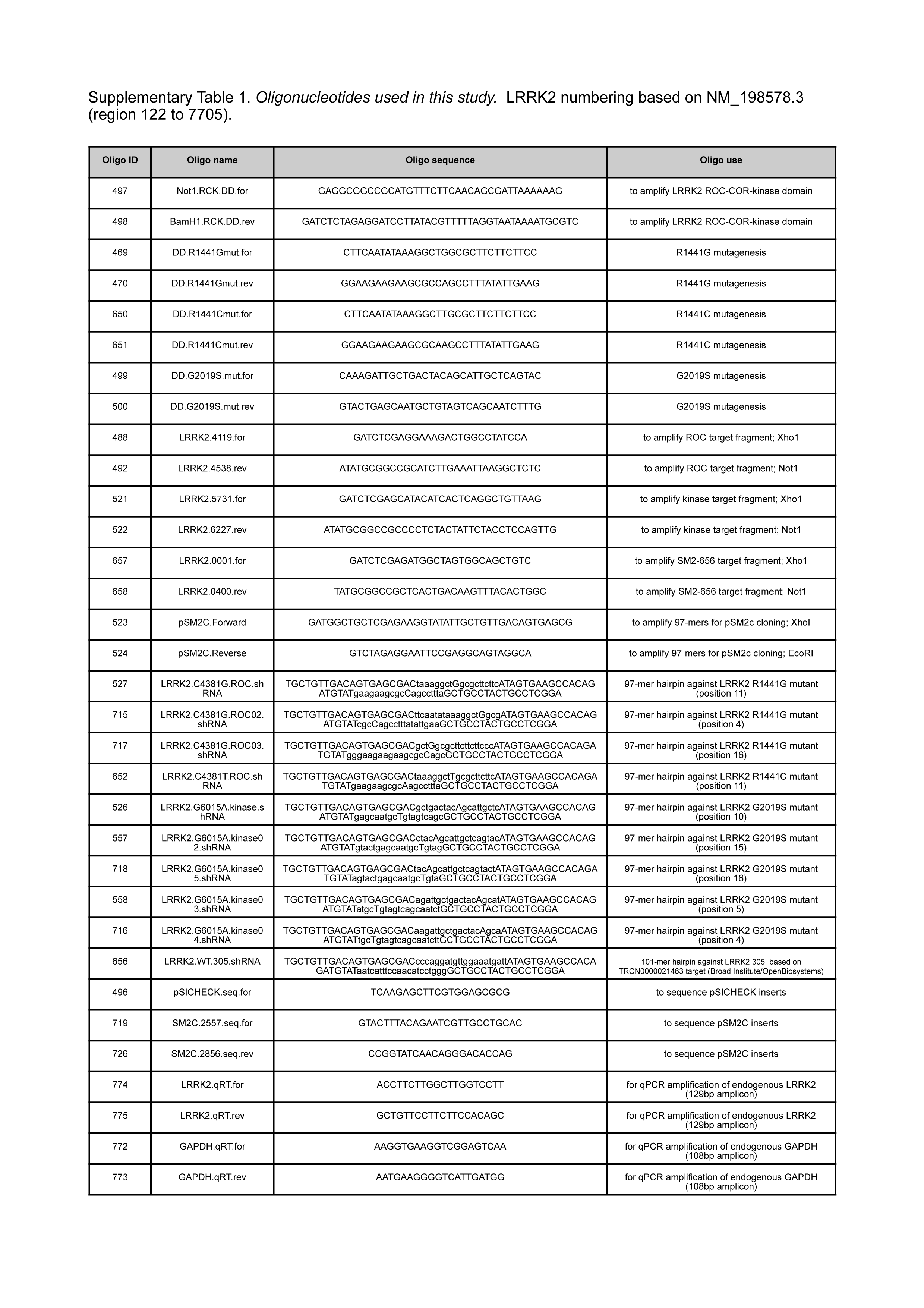

Supplement: Table S1 — Oligonucleotides used in this study. (TIF) [file pone.0021352.s001.tif]
